# Inactivation of uptake hydrogenase leads to enhanced and sustained hydrogen production with high nitrogenase activity under high light exposure in the cyanobacterium *Anabaena siamensis* TISTR 8012

**DOI:** 10.1186/1754-1611-6-19

**Published:** 2012-10-10

**Authors:** Wanthanee Khetkorn, Peter Lindblad, Aran Incharoensakdi

**Affiliations:** 1 Laboratory of Cyanobacterial Biotechnology, Department of Biochemistry, Faculty of Science, Chulalongkorn University, Bangkok, 10330, Thailand; 2 Photochemistry and Molecular Science, Department of Chemistry – Ångström Laboratory, Uppsala University, Box 523, SE-75120, Uppsala, Sweden

**Keywords:** *Anabaena siamensis*, Heterocyst differentiation, *HupS* inactivation, Hydrogen production, Nitrogenase activity, Uptake hydrogenase

## Abstract

**Background:**

Biohydrogen from cyanobacteria has attracted public interest due to its potential as a renewable energy carrier produced from solar energy and water. *Anabaena siamensis* TISTR 8012, a novel strain isolated from rice paddy field in Thailand, has been identified as a promising cyanobacterial strain for use as a high-yield hydrogen producer attributed to the activities of two enzymes, nitrogenase and bidirectional hydrogenase. One main obstacle for high hydrogen production by *A. siamensis* is a light-driven hydrogen consumption catalyzed by the uptake hydrogenase. To overcome this and in order to enhance the potential for nitrogenase based hydrogen production, we engineered a hydrogen uptake deficient strain by interrupting *hupS* encoding the small subunit of the uptake hydrogenase.

**Results:**

An engineered strain lacking a functional uptake hydrogenase (*∆hupS*) produced about 4-folds more hydrogen than the wild type strain. Moreover, the *∆hupS* strain showed long term, sustained hydrogen production under light exposure with 2–3 folds higher nitrogenase activity compared to the wild type. In addition, HupS inactivation had no major effects on cell growth and heterocyst differentiation. Gene expression analysis using RT-PCR indicates that electrons and ATP molecules required for hydrogen production in the *∆hupS* strain may be obtained from the electron transport chain associated with the photosynthetic oxidation of water in the vegetative cells. The *∆hupS* strain was found to compete well with the wild type up to 50 h in a mixed culture, thereafter the wild type started to grow on the relative expense of the *∆hupS* strain.

**Conclusions:**

Inactivation of *hupS* is an effective strategy for improving biohydrogen production, in rates and specifically in total yield, in nitrogen-fixing cultures of the cyanobacterium *Anabaena siamensis* TISTR 8012.

## Introduction

The N_2_-fixing cyanobacterium *Anabaena siamensis* TISTR 8012, a novel strain isolated from rice paddy field in Thailand has been reported to have a high potential for hydrogen production with the ability to utilize sugars as substrate to produce hydrogen [1]. In *Anabaena*, there may be three enzymes directly involved in hydrogen metabolism. 1) Nitrogenase, a multiprotein enzyme complex consisting of two proteins, dinitrogenase (MoFe protein), encoded by *nifD* and *nifK*, and the dinitrogenase reductase (Fe protein), encoded by *nifH*. This enzyme catalyzes the reduction of atmospheric N_2_ to ammonia as well as the reduction of proton (H^+^) to hydrogen [2,3]. In the absence of the substrate N_2_, nitrogenase may exclusively catalyze hydrogen production. 2) Uptake hydrogenase, a heterodimeric enzyme with at least two subunits, HupS (small subunit) and HupL (large subunit). The large subunit, encoded by *hupL*, contains the active site, consisting of four conserved cysteine residues that are involved in the coordination of the metallic NiFe at center of the active site. The small subunit, encoded by *hupS*, contains three FeS clusters which have a function in transferring electrons from active site of HupL to the electron transport chain. The physiological function of the uptake hydrogenase is recycling of hydrogen produced by nitrogenase [2-5]. 3) Bidirectional hydrogenase, a heteropentameric, NAD^+^-reducing enzyme, encoded by *hoxEFUYH*. It consists of two protein complexes; hydrogenase (HoxY and HoxH) and a diaphorase unit (HoxE, HoxF and HoxU). The bidirectional hydrogenase is commonly found, though not universal, in both N_2_-fixing and non-N_2_-fixing cyanobacteria and catalyzes both consumption and production of molecular hydrogen [2,3,6].

In *A. siamensis*, an enhanced hydrogen production is mainly achieved through the nitrogenase enzyme [7]. However, the net hydrogen yield is lost due to the activity of the uptake hydrogenase. To overcome this, we engineered a hydrogen uptake deficient strain by interrupting *hupS* with an antibiotic resistance cassette. Previous studies have reported that N_2_-fixing cyanobacteria such as *Nostoc punctiforme*, *Anabaena* sp. strain PCC 7120, *Anabaena variabilis* and *Nostoc* sp. strain PCC 7942 with inactivated uptake hydrogenases show an ability to produce hydrogen at higher rate when compared to their corresponding wild type strains [8-12]. Interestingly, previous reports mainly focused on HupL inactivation since the active site of uptake hydrogenase is located in the large subunit. Therefore, we focused on HupS in *A. siamensis* TISTR 8012. The structural *hupS* and *hupL* genes of *A. siamensis* have been identified and sequenced [13]. *hupS* is located upstream of *hupL* and the predicted gene products for *hupS* and *hupL* consist of 320 and 531 amino acids, respectively. Their deduced amino acid sequences show higher than 90% and 80% similarity for HupS and HupL, respectively when compared to other cyanobacteria [13]. RT-PCR analysis revealed that *hupS* and *hupL* were co-transcribed with an enhanced transcription when the cells were grown under N_2_-fixing condition [13]. HupS and HupL of *A. siamensis* and other cyanobacteria need to go through a maturation process to become a fully functional enzyme [14].

Thus, in the present study we engineered a strain lacking a functional uptake hydrogenase (*∆hupS*) with the aim to enhance hydrogen production in *A. siamensis* TISTR 8012. In addition, the nitrogenase activity and transcript levels of genes involved in hydrogen metabolism and photosynthetic pathways in the *∆hupS* strain were investigated. As expected, the *∆hupS* strain was more efficient in hydrogen production under long term of light exposure than the wild type strain and the production could be prolonged for more than 72 h under light conditions.

## Results and discussion

### The confirmation on a complete segregation of a *∆hupS* strain of *Anabaena siamensis*

**Figure 1:**
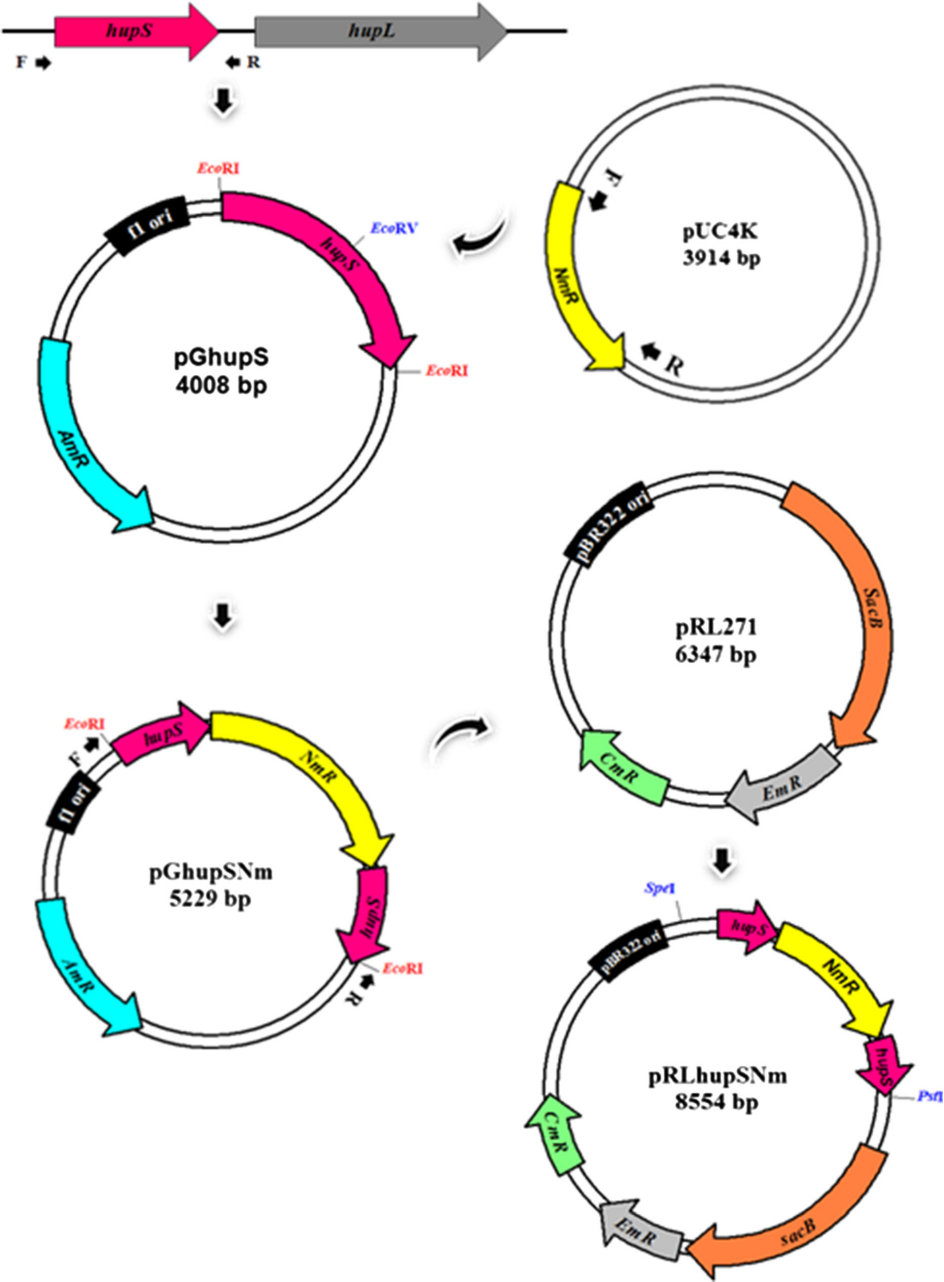
**Strategy for the construction of a recombinant plasmid containing an interruption in *hupS *(*∆hupS*).** Details as described in Materials and methods.

**Figure 2:**
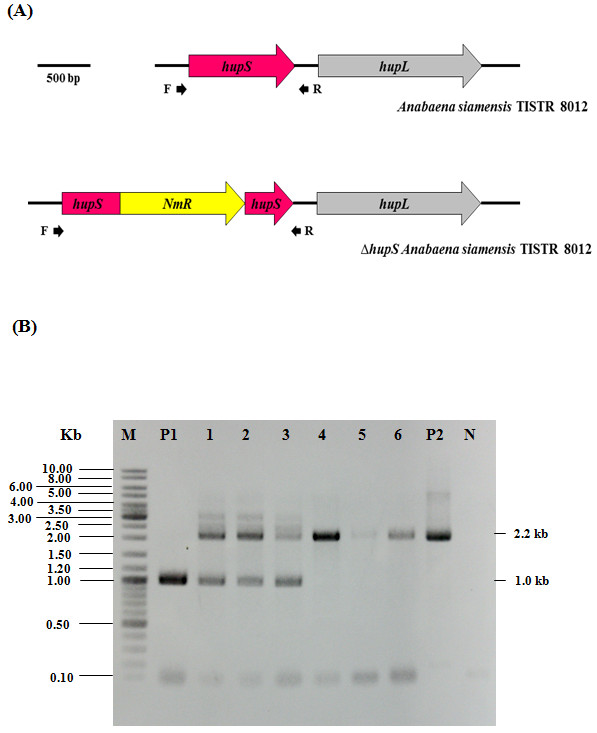
**Engineering of a *∆hupS *strain of *Anabaena siamensis *TISTR 8012.** (**A**) Physical map of *hupSL* in wild type *A. siamensis* TISTR 8012 and an engineered strain lacking a functional uptake hydrogenase (*∆hupS*) created by interrupting *hupS* with neomycin antibiotic resistant cassette. (**B**) Confirmation of complete segregation of *∆hupS* was performed by using colony PCRs and analyzed by 0.8% agarose gel electrophoresis. Primer pairs specific to *hupS* was used; Lane M: GeneRuler^TM^ DNA ladder (Fermentas), Lane P1: Positive control (PCR product using genomic DNA of wild type as template), Lanes 1–3: PCR products of recombinant colonies cultured in BG11 medium in which the addition of antibiotic for 2 weeks did not show complete segregation, Lanes 4–6: PCR products of recombinant colonies cultured in BG11medium in which the addition of antibiotic for 4 weeks did show complete segregation, Lane P2: Positive control (PCR product using pRLhupSNm plasmid as template), Lane N: Negative control using H_2_O as template.

After transformation of recombinant plasmid (pRLhupSNm) into cells of *Anabaena siamensis* TISTR 8012 (Figure 1), recombinant colonies were selected on BG11 plate containing 25 ug mL^-1^ of neomycin and transferred to BG11 broth containing antibiotic at the same concentration before analyzing for complete segregation using colony PCRs. To ensure complete segregation of *∆hupS* cells, colony PCRs were performed by using a primer pair specific to *hupS* as shown in Figure 2A. The results show that PCR products obtained from different recombinant colonies after two weeks did not show complete segregation whereas the completely segregated recombinant strains were found after 4 weeks of cultivation (Figure 2B). A completely segregated recombinant strain was selected for further analysis.

### Effect of *hupS* inactivation on hydrogen production, growth rate and heterocyst differentiation

**Figure 3:**
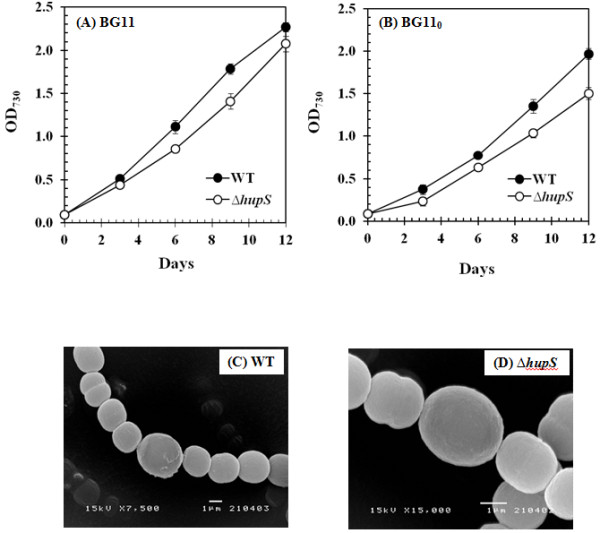
**Characterization of wild type and *∆hupS *strains of *Anabaena siamensis *TISTR 8012.** Comparison of growth rate between wild type and the *∆hupS* strains when cells were grown in either BG11 (**A**) or BG11_0_ (**B**) medium, means ± S.D. (n=3). Error bars are included in the graphs, some may be smaller than the symbols used. The morphology of wild type (**C**) and *∆hupS* strain (**D**) of *A. siamensis* TISTR 8012 cells observed under Scanning Electron Microscope (SEM) grown without the addition of N-source (BG11_0_ medium).

The physiological characterization of the *∆hupS* strain of *A. siamensis* TISTR 8012 was investigated by comparision with the corresponding wild type strain. *A. siamensis* TISTR 8012 wild type and *∆hupS* strain were grown in media with combined N-source (BG11) and without N-source (BG11_0_). Samples were taken to measure the optical density of cell culture every three days of cultivation. The results showed that even though the growth rate of the wild type and *∆hupS* strains had a similar pattern in both media the *∆hupS* strain grew slightly slower than the wild type strain (Figure 3A, B), suggesting that HupS inactivation had only minor effects on cell growth. This is in agreement with earlier observations using other filamentous cyanobacterial strains [8-12]. Moreover, under N_2_-fixing condition there was no discernible difference in physiological morphology between the *∆hupS* and wild type strains when observed under the Scanning Electron Microscope (SEM) (Figure 3C,D) and the heterocyst frequency in the *∆hupS* filaments gradually increased with time in a similar manner to that in the wild type (data not shown).

**Figure 4:**
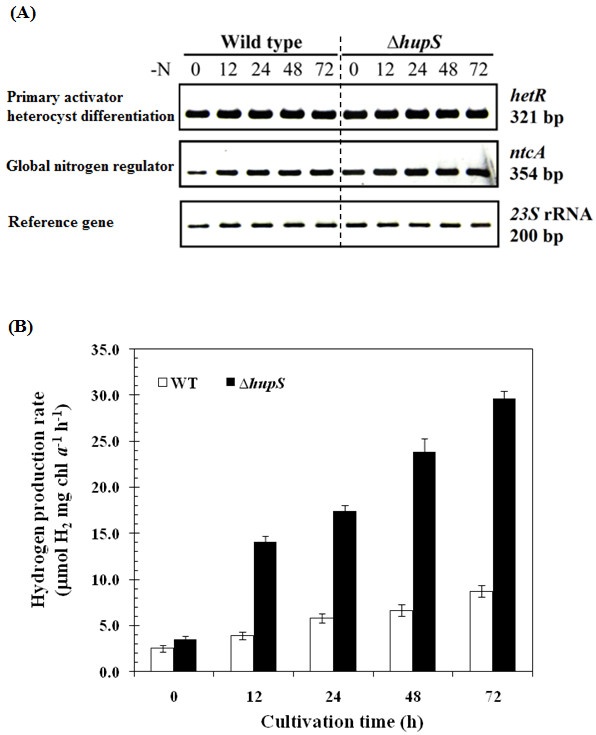
**Effect of HupS inactivation on genes transcription and hydrogen production.** Comparison of transcription levels of *hetR* and *ntcA* genes (**A**) and hydrogen production (**B**) between wild type and *∆hupS* strains of *Anabaena siamensis* TISTR 8012 when grown in BG11_0_ medium for various times at 40 μEm^-2^s^-1^. Hydrogen production rate was determined under continuous illumination of 40 μEm^-2^s^-1^ and anaerobic condition for 12 h. Means ± S.D. (n=3).

Furthermore, the transcription levels of key genes involved in heterocyst differentiation, *ntcA* and *hetR,* were examined under N_2_-fixing conditions by RT-PCR. The transcription factor NtcA encoded by *ntcA*, is a key transcriptional factor required for the activation of many genes involved in nitrogen and carbon metabolism [15,16]. In addition, NtcA is also required for the development and function of mature heterocysts. HetR, a serine-type protease encoded by *hetR*, is expressed early during heterocyst differentiation and is crucial to the differentiation process [17]. Mutation in the *hetR* inhibits early steps in the formation of heterocysts while over-expression of *hetR* gives rise to multiple heterocysts [18]. Both NtcA and HetR are auto-regulated [19,20]. The experiments revealed no discernible differences in either *ntcA* or *hetR* transcript levels between *∆hupS* and wild type strains after 72 h cultivation (Figure 4A). This suggests that HupS is not essential for heterocyst differentiation.

When analyzing hydrogen production, the wild type and *∆hupS* strains of *A. siamensis* TISTR 8012 were grown under N_2_-fixing conditions (BG11_0_ medium) for 12, 24, 48, and 72 h, respectively. Hydrogen production was then determined under continuous illumination of 40 μEm^-2^s^-1^ and anaerobic condition for 12 h. Interestingly, the *∆hupS* strain produced hydrogen at a significantly higher rate than that of the wild type (Figure 4B). The maximum hydrogen production rate of the *∆hupS* strain was 29.7 μmol H_2_ mg chl *a*^-1^h^-1^ when grown in BG11_0_ medium for 72 h, which is almost 4-folds higher than that observed in the wild type under normal growth condition. These results demonstrate that inactivation of *hupS* is an effective strategy for improving cyanobacterial photobiological hydrogen production in *A. siamensis* TISTR 8012. Similar observations have earlier been made in other filamentous cyanobacterial strains [8-12].

### Sustained hydrogen production and enhanced nitrogenase activity under long term of light exposure in the ∆*hupS* strain

**Figure 5:**
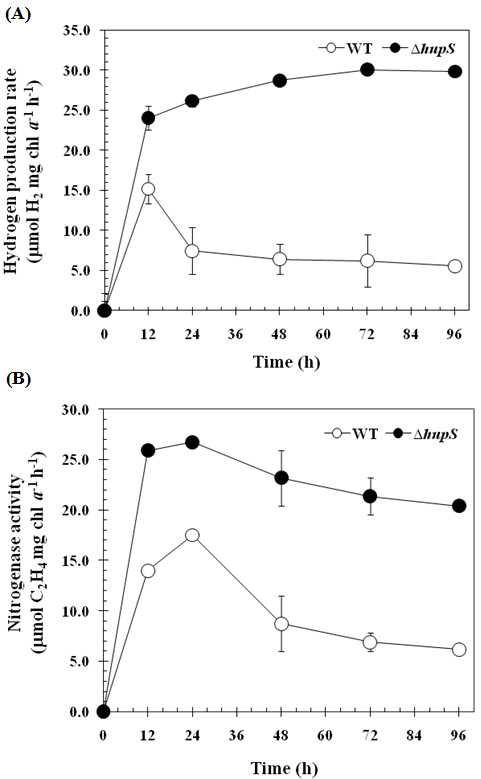
**Effect of HupS inactivation on the sustainability of hydrogen production and nitrogenase activity.** Comparison of hydrogen production (**A**) and nitrogenase activity (**B**) between wild type and *∆hupS* strains of *Anabaena siamensis* TISTR 8012 was done after growth in BG11_0_ medium for 4 days at 40 μEm^-2^s^-1^. The collected cells were determined for hydrogen production and nitrogenase activity after incubation for various times under continuous illumination of high light intensity at 200 μE m^-2^s^-1^ and anaerobic condition. Means ± S.D. (n=3).

We have previously reported that hydrogen production of wild type cells of *A. siamensis* TISTR 8012 decreased when the cells during the production phase were subject to long duration of high light intensity exposure (200 μE m^-2^s^-1^) with the consequence of high uptake hydrogenase activity [1]. Therefore, the *∆hupS* strain was grown under N_2_-fixing condition followed by measuring light dependent hydrogen production for various times up to 96 h. Interestingly, the *∆hupS* strain showed much higher hydrogen production under long term light exposure than the wild type strain (Figure 5A). The hydrogen production rate in the *∆hupS* strain continued to increase even after exposure to light longer than 12 h and the production could be prolonged up to 96 h under high light conditions. The nitrogenase activity of the *∆hupS* strain was also investigated under the same conditions. The *∆hupS* strain showed increased nitrogenase activity upon incubation up to 96 h with maximum activity detected after 24 h (Figure 5B). In contrast, the wild type strain had about 2–3 fold lower nitrogenase activity than the *∆hupS* strain. A previous study in *A. variabilis* mutant strain AVM13 in which *hupL* was interrupted showed no difference of nitrogenase activity when compared to wild type strain. Moreover, the higher hydrogen production by the AVM13 strain under N_2_-fixing conditions was not sustainable and a dramatically decreased hydrogen production was detected after 30 h incubation [8]. Our results demonstrate that the *∆hupS* strain of *A. siamensis* TISTR 8012 has a high potential for hydrogen production and an ability for long term, sustained hydrogen production under light exposure.

### Transcription levels of genes related to hydrogen metabolism and photosynthesis in wild type and ∆*hupS*

**Figure 6:**
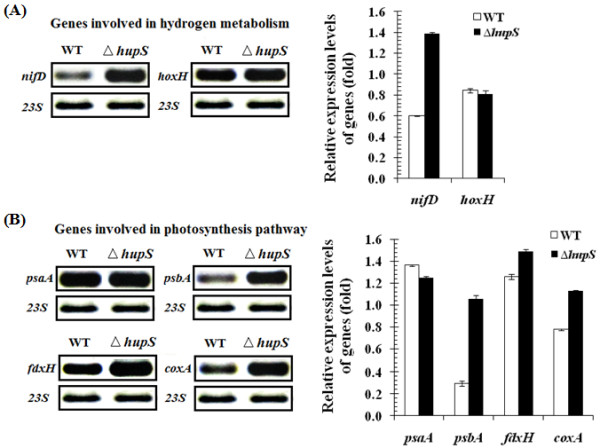
**Transcript analyses of genes involved in hydrogen metabolism (A) and photosynthesis pathway (B).** RT-PCR using total RNA isolated from wild type and *∆hupS* cells grown in BG11_0_ medium without N-source for 12 h. The PCR amplification using cDNAs of respective genes were performed using specific primers. *23S* rRNA was used as control when determining the relative level of the respective transcript and the intensities of the PCR generated DNA fragments were determined by using GeneTools program.

The *∆hupS* strain was examined for the relative transcript levels of genes encoding proteins involved in hydrogen metabolism and the photosynthesis pathway in order to provide important information for the regulation of hydrogen metabolism as affected by *hupS* inactivation. Significantly enhanced *nifD* but not *hoxH* transcript levels under N_2_-fixing conditions were observed in the *∆hupS* strain (Figure 6A). This indicates that the increased hydrogen production in the *∆hupS* strain may largely be due to higher nitrogenase activity with little or no contribution from the bidirectional hydrogenase. However, since the uptake hydrogenase which is one of the enzymes to supply electrons to the nitrogen fixation process is inactivated, the nitrogenase is likely to receive ATP and reducing equivalents from other pathways. The transcript levels of *psbA,* encoding the D1 protein of photosystem II, and *fdxH*, encoding a heterocyst-specific ferredoxin mediating electron transport to the nitrogenase in heterocysts, were significantly up-regulated in the *∆hupS* strain (Figure 6B).

This may suggest that electrons and ATP needed for hydrogen production in the *∆hupS* strain of *A. siamensis* TISTR 8012 can be obtained from the electron transport chain associated with the photosynthetic oxidation of water of photosystem II in the vegetative cells. In addition, there was no change observed in the transcription level of the *psaA* encoding the core protein of photosystem I (Figure 6B). It should be noted that the source of electron transfer to nitrogenase could arise from not only vegetative cells but also from within the heterocysts. Previously, proteins involved in the oxidative pentose phosphate pathway have been reported to be more abundant in heterocysts of a hydrogen uptake deficient strain of *Nostoc punctiforme*[21].

The inactivation of HupS of *A. siamensis* TISTR 8012 (*∆hupS*) resulted in a significant up-regulation of *coxA* encoding the cytochrome c oxidase subunit I which is present in vegetative cells only [22]. The increase of CoxA activity would lower the level of O_2_ in vegetative cells resulting in less inhibition of bidirectional hydrogenase, leading to enhanced hydrogen production. Nevertheless, to further explore the effect of HupS inactivation on cell metabolism, the global protein expression level should be investigated.

### Growth competition of wild type and ∆*hupS* strains in a mixed culture

**Figure 7:**
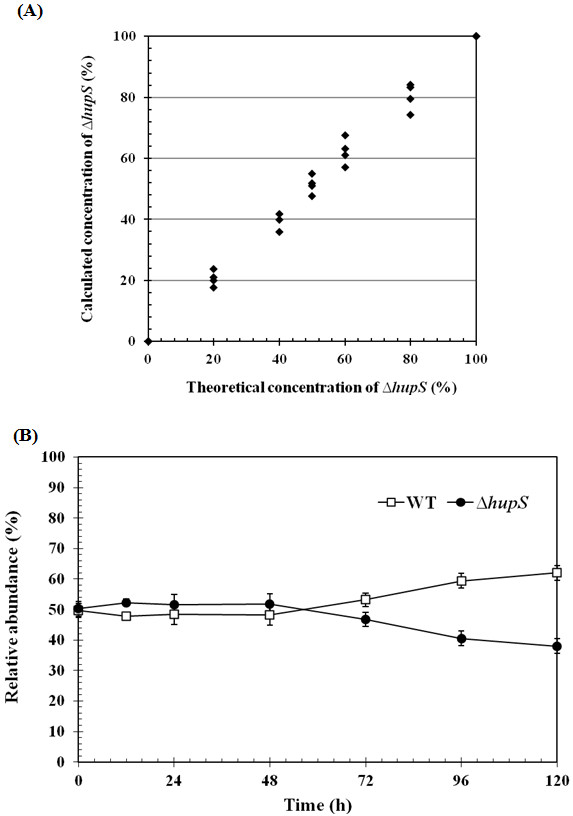
**The ability of the *∆hupS *strain to compete with the wild type strain analyzed using a molecular method to determine the relative abundances of the strains in mixed cultures.** (**A**) Standard curve, theoretical and calculated % of *∆hupS* strain with respect to wild type in mixed cultures. (**B**) The relative abundance of wild type and *∆hupS* strains. The relative abundance at time = 0 was set at 50% (ratio of wild type to *∆hupS* strain was 1:1).

The ability of the *∆hupS* strain of *A. siamensis* TISTR 8012 to compete with the wild type in a mixture without the addition of an antibiotic was studied using a molecular method to analyze the relative abundances of the two strains in a single sample. To demonstrate the possibility to use this method to quantify the relative abundance of the wild type and *∆hupS* strains, axenic cultures of wild type and *∆hupS* strains were mixed in known proportions before performing colony PCRs using primers specific to *hupS*. Obtained DNA fragments were analyzed by 0.8% agarose gel electrophoresis, their band intensities were quantified resulting in a standard calibration curve (Figure 7A). The method was then applied to an experiment analyzing the growth of the wild type and *∆hupS* strains in a mixed culture for 120 h. After around 50 h in the 50:50 mixed culture, the wild type started to grow on the relative expense of the *∆hupS* strain (Figure 7B), in agreement with an earlier observation using a *∆hupL* strain of *Anabaena*/*Nostoc* PCC 7120 [23]. The mixed culture of wild type and ∆*hupS* strains reached a ratio of 60:40 after 96 h. It is worth noting that the *∆hupS* strain may grow in antibiotic free medium without reverting back to wild type. However, the long term stability of the engineered strain should be further established in long term, large scale hydrogen production facilities.

## Conclusions

This study further explores the potential for solar based biohydrogen production using a purpose designed engineered cyanobacterial strain, *∆hupS*, using a general strategy outlined elsewhere [24]. We demonstrate that inactivation of *hupS* encoding the small subunit of the uptake hydrogenase in the cyanobacterium *Anabaena siamensis* TISTR 8012 results in long term sustainable, light dependent hydrogen production with enhanced nitrogenase activity and only minor effects on cell growth and heterocyst differentiation when compared with the wild type strain. The *∆hupS* strain was found to compete well with the wild type up to 50 h in a mixed culture. However, the effects of HupS inactivation on general and specific metabolism as well as the long term stability of the *∆hupS* strain warrant further investigations.

## Materials and methods

### Strain and growth conditions

The N_2_-fixing cyanobacterium *Anabaena siamensis* TISTR 8012 cells were grown in 50 mL of BG11_0_ medium (without N-source) and BG11 medium containing 18 mM NaNO_3_ as N-source [25], both media were buffered with 20 mM HEPES-NaOH (pH 7.5). For *∆hupS* strain, cells were grown in either BG11 or BG11_0_ media containing 25 μg mL^-1^ neomycin antibiotic. The initial cell concentration was adjusted to an OD_730_ of 0.1 and cultures were incubated aerobically under continuous illumination of 40 μEm^-2^s^-1^ with cool white fluorescent lamps from two sides on a rotatory shaker at 160 rpm and 30°C. The growth rate was monitored by measuring the optical density of the culture at 730 nm with a spectrophotometer. The total amount of chlorophyll *a* (chl *a*) was determined spectrophotometrically at 665 nm in 90% (v/v) methanol extracts [26]. For the morphological study, *A. siamensis* TISTR 8012 cells were observed under Scanning Electron Microscope, SEM (JEOL model JSM-5410LV, Japan).

### Construct of recombinant plasmid and conjugative gene transfer

The strategy for construction of recombinant plasmid containing target gene interruption could be divided into three steps as shown in Figure 1. The first step, the *hupS* gene sequence information in *A. siamensis* TISTR 8012 was obtained from the NCBI database, accession number AY152844. *hupS* was amplified from extracted genomic DNA of *A. siamensis* TISTR 8012 cells by using specific primers, HupSF2 (gcatgcatgactaacgtactctggct) and HupSR2 (gcatgcgtctccattcccattaccta). The obtained *hupS* PCR product was purified and ligated into the pGEM-T easy vector (Promega), creating pGhupS plasmid. The second step, the *Mlu*I fragment containing a neomycin (*NmR*) resistant cassette gene from pUC4K vector was modified blunt-ending and then inserted into *Eco*RV site within the *hupS* gene of the pGhupS plasmid to produce pGhupSNm plasmid. In the last step, a hupSNm fragment from pGhupSNm plasmid was amplified by using specific primers and then cloned into pRL271 vector to produce pRLhupSNm plasmid. pRLhupSNm plasmid functions as a cargo plasmid suitable to be transferred into *A. siamensis* TISTR 8012 cell. All plasmids were checked and confirmed by sequencing.

The cargo plasmid, pRLhupSNm was transformed into *E. coli* HB101 carrying the helper plasmid pRL623 and transferred to *A. siamensis* TISTR 8012 cell with the help of the conjugative plasmid pRL443 as shown in Table 1 by using the triparental mating method [27]. Single recombinant exconjugant colonies were selected on BG11 plate containing neomycin antibiotic at concentration of 25 μg mL^-1^. To ensure the complete segregation, obtained gene knockout was analyzed by colony PCRs.

**Table 1:** Plasmids used in this study

**Plasmids**	**Relevant characteristic(s)**	**Source / Reference**
pGEM-T easy	Cloning vector, Apr *lacZ*^′^*, mcs*	Promega
pRL271	Cloning vector carrying *sacB*, Em and Cm	GenBank accession #L05081
pUC4K	Source of Nm cassette	Amersham
pRL632	Helper plasmid carrying metylates *AvaI*, *AvaII* and *AvaIII* sites	[27]
pRL443	Conjugative plasmid, Km spontaneous mutant of RK2	[27]
pGhupS	pGem-T easy vector contained *hupS*	This study
pGhupSNm	Nm cassette inserted into *Eco*RV site within *hupS* of pGhupS	This study
pRLhupSNm	Cloning vector, Apr *lacZ*^′^*, mcs*	This study

### Hydrogen production determination

The cells were harvested and resuspended in 5 mL medium in a 13 mL of glass vial, then sealed with a rubber septum and a proper screw lid. The vial was bubbled with argon gas for 15 min to eliminate oxygen and incubated under different conditions at 30°C before determining hydrogen production. After 12 h incubation, a 400 μL of head space gas sample was withdrawn from the vial with a gas tight syringe and the hydrogen gas was analyzed by a gas chromatograph (Peri-chrom PR2100, France) with a Molecular Sieve 5A 60/80 mesh column equipped with a thermal conductivity detector and argon as the carrier gas. The hydrogen production rate was expressed as μmol H_2_ mg chl *a*^-1^ h^-1^.

### Nitrogenase activity determination

*In vivo* nitrogenase activity was measured using the acetylene-reduction assay. In the absence of N_2_, the enzyme catalyzes the conversion of acetylene (C_2_H_2_) to ethylene (C_2_H_4_) gas. The reaction was carried out in a glass vial by incubation of the cells suspension (2 mL) with 1 mL of 10% (v/v) acetylene (C_2_H_2_) balanced in argon. The ethylene (C_2_H_4_) production was detected by using a Gas Chromatograph with a Porapak Q, 50/80 mesh column equipped with a flame ionization detector (Shimadzu, Japan). Enzyme activity was expressed as μmol C_2_H_4_ mg chl *a*^-1^ h^-1^.

### Transcription analysis

The total RNA was extracted from cells in each condition by using the TRI Reagent® and treated with DNase (Fermentas) for DNA digestion. The treated RNA (1 μg) was converted to single stranded cDNA with the iScript^TM^ cDNA Synthesis Kit (Bio-RAD), according to the manufacturer’s instruction. RT-PCR amplifications using cDNAs of the respective genes were performed using corresponding primers. Negative controls for the RT-reaction were RT-PCR on DNaseI treated RNA without RT-enzyme. Negative controls for the PCR reactions were PCR amplification without cDNA added and positive controls were performed with genomic DNA using the corresponding primers. All primers used are listed in Table 2. The PCR conditions consisted of 95°C for 3 min, followed by 30 cycles of 95°C for 15 sec, 50°C for 20 sec and 72°C for 20 sec, and then a final extension at 72°C for 3 min. The PCR product was analyzed by 1.0% (w/v) agarose gel electrophoresis.

**Table 2:** Primers used in RT-PCR reactions

**Primer**	**Sequence 5**^′^**to 3**	**Target of primer pair**	**PCR product, bp**
ASnifDF1	tcgtattcggtggtgacaaa	*nifD*	204
ASnifDR1	gagacacaccacggaaacct		
AShoxHF1	gaatccgtctgcgtcaattt	*hoxH*	284
AShoxHR1	gcaaatgtccgtcgtaggtt		
23F	gctaagcgatgtaccgaagc	*23S* rDNA	200
23R	taacccagagtggacgaacc		
PsaAF1	ctgttgaaaggtgtattgtt	*psaA*	489
PsaAR1	aggagctaccttcagtttat		
PsbAF1	gcacattcaactttatgatt	*psbA*	390
PsbAR1	ccaaaattgagttattgaag		
FdxHF1	atggctagctaccaagttag	*fdxH*	299
FdxHR1	ttaagcaaggtacggttctt		
CoxAF1	gcgagattacttcagtttta	*coxA*	426
CoxAR1	atccaaataccttctcctac		
NtcAF1	cgagtctactttcttttgaa	*ntcA*	354
NtcAR1	aaaatcacgacagagaatta		
HetRF	ggatgaccggacatttgcac	*hetR*	321
HetRR	ccataagcgatcgcaagagg		

### Determination of the relative abundance of wild type and ∆*hupS* strain of *Anabaena siamensis* TISTR 8012 in a mixed culture

Axenic cultures of *A. siamensis* TISTR 8012 wild type and the *∆hupS* strain were mixed in known proportions. Colony PCRs were performed using primers specific to *hupS* and analyzed by 0.8% agarose gel electrophoresis. The sizes of the obtained PCR product were approximately 1.0 kb and 2.2 kb representing *hupS* of the wild type and *hupS* interrupted with neomycin resistant cassette gene of the engineered strain, respectively. The intensities of the PCR bands were compared within each lane and calculated by using GeneTools program (SynGene, USA) to detect the presence and the relative abundance of the wild type and *∆hupS* strain in a single sample. Using this protocol a standard curve was generated and shown as percent of *∆hupS* with respect to wild type strain. For competition experiments, the axenic cultures of wild type and *∆hupS* strains of *A. siamensis* TISTR 8012 were mixed together in a 50 mL growth flask in a ratio of 1:1 based on OD_730_ measurements and incubated under growth conditions. The relative abundance of the wild type versus the *∆hupS* strain was then calculated during the experiment.

## Competing interest

The authors declare that they have no competing interests.

## Authors' contributions

WK, PL and AI designed the study, analyzed the data and wrote the manuscript. WK performed the experiments. All authors read and approved the final manuscript.
